# Small Strokes, Big Impact: Excessive Mortality After Acute Ischemic Stroke in Parkinson's Disease

**DOI:** 10.1002/mds.70218

**Published:** 2026-02-09

**Authors:** Lee E. Neilson, Chen Lin, Anusha Mishra, Gregory D. Scott

**Affiliations:** ^1^ Department of Neurology Oregon Health & Science University Portland Oregon USA; ^2^ Neurology and Research Service VA Portland Health Care System Portland Oregon USA; ^3^ Department of Neurology University of Iowa Iowa City Iowa USA; ^4^ Department of Neurology Birmingham VA Medical Center Birmingham Alabama USA; ^5^ Department of Neurology Louisiana State University Health‐Shreveport Shreveport Louisiana USA; ^6^ Department of Pathology and Laboratory Services VA Portland Medical Center Portland Oregon USA; ^7^ Department of Pathology Oregon Health & Science University Portland Oregon USA

**Keywords:** ischemic stroke, metabolism, mortality, Parkinson's disease, transient ischemic attack, vascular health

## Abstract

**Background:**

Acute ischemic stroke (AIS) in those with Parkinson's disease (PD) is a growing healthcare burden but mortality studies are significantly lacking.

**Objective:**

To map short‐ and long‐term survival, stratified by relevant comorbidities and stroke characteristics.

**Methods:**

Individuals from the United States Veterans Health Administration were included. AIS and PD were defined by validated International Classification of Diseases (ICD) codes, pharmacy data, and encounter type. Covariates included demographics, smoking status, cardiovascular comorbidities, post‐traumatic stress disorder, traumatic brain injury, and frailty. Individuals with PD and stroke were matched 4:1 to controls and survival was measured over 10 years. Two percent of strokes were manually reviewed for size, location, and mechanism per TOAST (Trial of Org 10172 in Acute Stroke Treatment) criteria.

**Results:**

A total of 3173 cases of PD and AIS were identified and matched to groups of PD‐only, stroke‐only, and controls (N = 12,692 each). Survival analysis showed PD‐only and stroke‐only had a predictable increased mortality (added deaths ranged from 1.9 [0.9, 2.9] to 4.7 [2.9, 6.6] per 100 person‐years). PD + stroke showed further increased mortality with distinct timelines at 0–1‐, 1–5‐, and 5–10‐year periods and synergy accounted for 2.9 [0.5, 5.4] to 19.3 [14.0, 24.6] additional deaths across 10 years. Effects persisted after matching for cardiovascular comorbidities, neurotrauma, and frailty. PD + stroke compared with stroke‐only had smaller strokes (80.0% vs. 63.2%) and no differences in location, mechanism, or recurrence rates.

**Conclusions:**

Patients with PD and AIS had greater than expected mortality that was independent of comorbidities or stroke characteristics. This may reflect widespread structural and functional deficits that may warrant a targeted therapeutic strategy. © 2026 The Author(s). *Movement Disorders* published by Wiley Periodicals LLC on behalf of International Parkinson and Movement Disorder Society.

## 1 Introduction

Parkinson's disease (PD) is the second most common, but fastest growing, neurodegenerative disorder, with a doubling in the global burden of disease over the past 30 years.^1^ It is expected to increase over time, irrespective of geography and socioeconomic status.^2^ Similarly, stroke prevalence is growing and is the third leading cause of death and disability in the world.^3^ Amplifying this healthcare challenge is the fact that persons with PD have an increased risk of having a stroke.^4^, ^5^, ^6^

It has also been argued that PD's influence on stroke‐related mortality may be magnified. However, data are limited. One matched cohort study from Thailand found that PD reduced mortality in the first 4 weeks following stroke, but increased it thereafter.^7^ In contrast, a nested cohort study from Taiwan and a matched cohort study from Korea showed increased mortality in PD + stroke patients but failed to provide nuanced timing information.^4^, ^8^ Further limiting interpretation of these studies are significant methodological limitations: the ascertainment of PD relied exclusively on single International Classification of Diseases (ICD) codes without manual validation, key prognostic covariates were not measured (eg, race and smoking status), and the absence of a PD‐only and a negative control group did not permit disentanglement of interactions. Studies using single‐code PD case definitions are particularly challenging to interpret because positive predictive values (PPVs) are low outside of notes authored by movement disorder experts^9^ and misclassification of vascular parkinsonism may represent a significant source of bias in a stroke‐based study; one study reported a rate as high as 29% of false‐positives.^10^ Thus, it is unclear if and how PD and stroke interact in the short and long term, and if these effects are independent of large‐effect characteristics like age, race, and common comorbidities such as cardiovascular disease and diabetes. Understanding this intersection may elucidate the underlying biology of PD and may influence management of persons with PD, as vascular risk factor control is currently not considered a PD quality measure by the American Academy of Neurology.^11^

To fill these knowledge gaps, we tested the hypothesis that PD increases stroke‐related mortality. We designed a population‐based retrospective cohort study using the nationwide medical records database of the United States Department of Veterans Affairs (VA). This is an ideal database because it represents the largest source of integrated healthcare data in the United States (US) and because military veterans have high rates of both PD and stroke. Additionally, this database enables the use of previously validated PD definitions with high accuracy,^12^ allows longer follow‐up time and the inclusion of PD and stroke‐alone populations, and accounts for key baseline risk factors.

## 2 Methods

### 2.1 Study Design, Data, and Institutional Approval

A cohort study design was used to measure mortality associated with PD and stroke. Data were obtained from the VA Corporate Data Warehouse (CDW) under institutional review board (IRB) approval (MIRB #04744) using a waiver of participant consent. All time periods were included in the study. Individuals were included if they were at least 40 years old as of October 1, 2021.

### 2.2 Definition of Cases and Variables

Within the CDW, PD was defined using previously validated criteria requiring appropriate diagnostic codes and medication and offers a PPV of 78.6%.^12^ Sensitivity analysis used a VA PD definition with a PPV of 90.0%, which required a PD diagnostic code to be documented in two encounters authored by a neurologist separated by a minimum of 1 year. Stroke was defined by having one or more ICD codes related to acute ischemic stroke (AIS): 362.3, 433, 434, 436 from ICD‐9 and/or H34.1, I63, I64 from ICD‐10. Transient ischemic attack (TIA) was similarly defined by ICD‐9 (431) and ICD‐10 (I61) codes.^13^ A 2% random sample of patients with AIS was manually reviewed for diagnostic accuracy and subsequently categorized based on stroke size, location, and presumptive mechanism according to TOAST (Trial of Org 10172 in Acute Stroke Treatment) criteria.^14^ PPV was 90.8%. Controls were matched 4:1 using propensity score matching^15^ (caliper width = 0.2) for age, sex, ethnicity, race, and smoking status.^16^ Missing demographic data were imputed using multiple imputation by chained equations.^17^ Index date was defined as the date of stroke – considered the date of the first relevant ICD code – and in groups without stroke it was defined as the same age (month and year) as the age of their matched case with stroke and PD. Two definitions were used for stroke recurrence: new inpatient stroke code after discharge from the initial stroke and any new inpatient stroke code occurring >30 days after the initial stroke.

### 2.3 Statistical Analyses

Survival analyses were performed over a 10‐year period and compared between groups with and without stroke and PD. Relative effects were measured by calculating hazard ratios (HR) using the Cox proportional hazards model. Absolute effects were measured using an Aalen additive hazards model.^18^ In the primary analysis, the groups were matched for birth year, age, sex, race, ethnicity, and smoking status. Three secondary survival analyses were then performed: (1) additional matching for VA frailty at the index date, (2) vascular comorbidities (including hypertension, coronary artery disease, hyperlipidemia, heart failure, atrial fibrillation, and diabetes), and (3) post‐traumatic stress disorder (PTSD) and traumatic brain injury (TBI). The VA frailty index is a validated summary measure of over 6000 diagnostic codes covering 31 disease entities (range: 0–1 where >0.2 is considered ‘frail’ and is associated with higher mortality).^19^ PD and stroke were removed before calculation of frailty at the index date. Synergy/positive interaction between joint effects of PD and stroke was defined as combined effects being greater than the sum of individual effects^20^ as per the Strengthening the Reporting of Observational Studies in Epidemiology (STROBE) statement.^21^ For analysis of recurrent stroke, cumulative incidence was evaluated after adjustment of competing risk of death. For stroke and cohort characteristics, chi square or analysis of variance (ANOVA) was performed to determine group differences in categorical and continuous variables, respectively. Analyses were conducted using RStudio (V4.4.1, Posit, Boston, MA, USA).

## 3 Results

### 3.1 Cohort Characteristics

A total of 3173 cases of individuals with PD and then a subsequent stroke (‘PD + Stroke’) were identified. Then 4:1 propensity score matching was completed based on age, sex, smoking status, race, and ethnicity for PD‐only, stroke‐only, and control groups (neither stroke nor PD). Baseline characteristics are shown in Table 1.

**TABLE 1:** Cohort characteristics

Characteristic	Control no PD, no stroke	Stroke only (no PD)	PD only (no stroke)	PD + stroke
Individuals (N)	12,692	12,692	12,692	3173
Birth year	1937.8 (±9.8)	1938.4 (±10.2)	1938.4 (±9.9)	1937.7 (±9.8)
Age (years)	76.9 (±8.4)	76.2 (±9.1)	76.3 (±8.6)	76.9 (±8.4)
Year of stroke	–	2014.9 (±4.6)	–	2014.7 (±4.6)
Sex				
Female	169 (1.3%)	143 (1.1%)	146 (1.2%)	44 (1.4%)
Male	12,523 (98.7%)	12,549 (98.9%)	12,546 (98.8%)	3129 (98.6%)
Smoking status				
Never	4854 (38.2%)	4711 (37.1%)	4811 (37.9%)	1213 (38.2%)
Current	2792 (22.0%)	2832 (22.3%)	2831 (22.3%)	700 (22.1%)
Former	5046 (39.8%)	5149 (40.6%)	5050 (39.8%)	1260 (39.7%)
Race				
American Indian or Alaska Native	72 (0.6%)	35 (0.3%)	51 (0.4%)	18 (0.6%)
Asian	77 (0.6%)	74 (0.6%)	113 (0.9%)	23 (0.7%)
Black or African American	1171 (9.2%)	1152 (9.1%)	1194 (9.4%)	295 (9.3%)
Native Hawaiian or other Pacific Islander	146 (1.2%)	91 (0.7%)	138 (1.1%)	33 (1.0%)
White	11,226 (88.4%)	11,340 (89.3%)	11,196 (88.2%)	2804 (88.4%)
Ethnicity				
Hispanic or Latino	863 (6.8%)	680 (5.4%)	977 (7.7%)	215 (6.8%)
Not Hispanic or Latino	11,829 (93.2%)	12,012 (94.6%)	11,715 (92.3%)	2958 (93.2%)
Secondary analysis: Before matching				
Total frailty index	0.11 (±0.11)	0.18 (±0.12)	0.21 (±0.12)	**0.28 (±0.13)**
Number of cardiovascular and lipid factors	1.97 (±1.55)	2.36 (±1.48)	2.72 (±1.40)	**2.87 (±1.34)**
Hyperlipidemia	5950 (46.9%)	8166 (64.3%)	8974 (70.7%)	**2313 (72.9%)**
Diabetes	3842 (30.3%)	5660 (44.6%)	5140 (40.5%)	**1594 (50.2%)**
Coronary artery disease	3593 (28.3%)	5782 (45.6%)	5192 (40.9%)	**1586 (50.0%)**
Atrial fibrillation	2026 (16.0%)	**3369 (26.5%)**	2551 (20.1%)	809 (25.5%)
Heart failure	1256 (9.9%)	2718 (21.4%)	2054 (16.2%)	**767 (24.2%)**
Hypertension	8362 (65.9%)	10,890 (85.8%)	10,650 (83.9%)	**2862 (90.2%)**
Dementia	1796 (14.2%)	3876 (30.5%)	5700 (44.9%)	**1833 (57.8%)**
Secondary analysis: After matching:				
Total frailty index	0.25 (±0.11)	0.25 (±0.11)	0.25 (±0.11)	0.26 (±0.12)
Number of cardiovascular and lipid factors	2.64 (±1.26)	2.78 (±1.26)	2.7 (±1.17)	2.87 (±1.34)
Hyperlipidemia	8788 (72.9%)	8705 (72.2%)	8802 (73.0%)	2195 (72.8%)
Diabetes	5626 (46.9%)	5578 (46.5%)	5655 (47.1%)	1411 (47.0%)
Coronary artery disease	5406 (44.8%)	5382 (44.6%)	5377 (44.6%)	1356 (45.0%)
Atrial fibrillation	2074 (17.3%)	2021 (16.9%)	1914 (16.0%)	526 (17.6%)
Heart failure	1769 (14.8%)	1718 (14.4%)	1589 (13.3%)	451 (15.1%)
Hypertension	10,834 (89.3%)	10,839 (89.4%)	10,852 (89.5%)	2707 (89.3%)
Dementia	4515 (39.8%)	4452 (39.3%)	4326 (38.2%)	1132 (40.0%)

*Note*: Bold type highlights the group with the greatest proportion of unmatched comorbidity.

Abbreviation: PD, Parkinson's disease.

### 3.2 Mortality, Timing, and Synergism of Stroke and PD

**FIG. 1:**
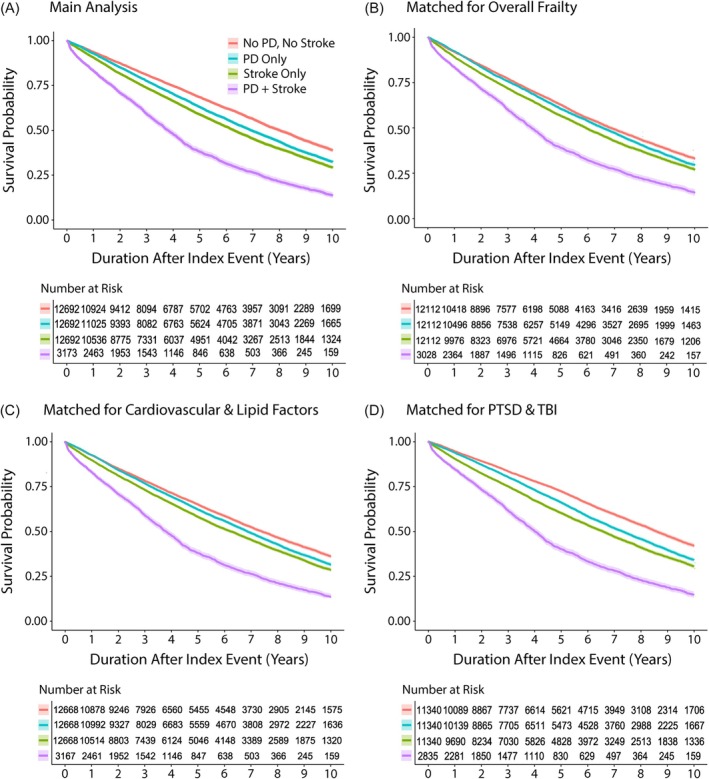
Impact of Parkinson's disease (PD) on survival after acute ischemic stroke. Survival probability is graphed for 10 years after the index date (date of stroke or equivalent age in controls) for groups: PD + Stroke (purple), PD‐only (cyan), stroke‐only (green), and controls (red). Panel (A) shows the main analysis of survival probability adjusting for sex, race, and smoking status. The subsequent panels show additional propensity matching for comorbidities: (B) frailty; (C) vascular comorbidities (hypertension, hyperlipidemia, coronary artery disease, heart failure, atrial fibrillation, diabetes mellitus); and (D) post‐traumatic stress disorder (PTSD) and traumatic brain injury (TBI). Risk tables for each group at yearly intervals are included under each graph. [Color figure can be viewed at wileyonlinelibrary.com]

Survival was lowest in the PD + stroke group (Fig. 1A). Cumulative incidence of death at 10 years was 61% in the control group, 70% in the stroke‐only group, 67% in the PD‐only group, and 86% in the PD + stroke group, representing an absolute excess mortality of 9%, 6%, and 25%, respectively, compared with controls. The time course of decreasing survival in the PD + stroke group occurred in three distinct stages. The PD + stroke group had a greater rate of decline in survival within the first year, again over the ensuing 5–6 years, and then a similar slope to stroke‐only and PD‐only groups up to 10 years. Survival analysis (number of added deaths per 100 person‐years) showed increased mortality in PD‐only (range 1.9 [0.9, 2.9] to 2.9 [2.2, 3.7]) and stroke (AIS)‐only groups (range 3.2 [2.1, 4.3] to 4.7 [2.9, 6.6]) (Table 2). A positive interaction between these two conditions with further increased mortality in the PD + stroke group was identified, and the combination accounted for additional deaths per 100 person‐years ranging from (2.9 [0.5, 5.4] to 19.3 [14.0, 24.6]) (Table 2). Analysis using a relative Cox proportional hazards model supported synergistic interaction of PD + stroke with HRs ranging from 2.31 [2.09, 2.57] to 4.50 [3.60, 5.62]. Analysis with higher PPV VA‐specific definition of PD recapitulated the results (Supplementary Fig. S1) with the PD + stroke group adding 2.7 [2.0, 3.4] deaths per 100 person‐years over the entire 10‐year period. When restricting the analysis to females only (which was a small subset in this VA‐based data), we observed similar trends, but no statistically significant differences were detected when using either the primary (Supplementary Fig. S3A) or high PPV (Supplementary Fig. S3B) definitions of PD.

**TABLE 2:** Death due to Parkinson's disease, stroke, transient ischemic attack, and interactions.

Parameter	Time interval						
	0–2 mo	2–4 mo	4–6 mo	6–12 mo	1–3 yr	3–5 yr	5–10 yr
AIS: added deaths per 100 person‐years [95% CI]							
PD‐only	–	–	–	1.9	2.3	2.9	3.8
	[−0.9, 2.2]	[−1.1, 1.8]	[−1, 2.1]	[0.9, 2.9]	[1.7, 2.9]	[2.2, 3.7]	[3.1, 4.5]
Stroke (AIS)‐only	4.7	3.9	3.3	3.2	3.3	3.2	3.3
	[2.9, 6.6]	[2.1, 5.6]	[1.6, 5.1]	[2.1, 4.3]	[2.7, 3.9]	[2.4, 3.9]	[2.6, 4.1]
Additional deaths from interaction of PD and stroke	19.3	8.7	6.0	2.9	4.2	8.7	4.2
	[14, 24.6]	[4.4, 13.2]	[1.8, 10.2]	[0.5, 5.4]	[2.8, 5.7]	[6.6, 10.9]	[2.1, 6.2]
TIA: added deaths per 100 person‐years [95% CI]							
TIA‐only	–	–	–	–	–	1.7	1.1
	[−2.3, −0.1]	[−1.7, 0.7]	[−0.2, 2.2]	[−0.1, 1.3]	[0.3, 1.2]	[1.1, 2.2]	[0.6, 1.6]
Additional deaths from interaction of PD and TIA	–	–	–	4.0	3.3	3.5	6.2
	[0.2, 5.8]	[1.7, 7.9]	[−1.6, 4.5]	[2.1, 5.9]	[2.2, 4.4]	[2, 4.9]	[4.7, 7.6]

*Note*: ‘–‘, not significant (*P* > 0.05); all other values significant at *P* < 0.05.

Abbreviations: mo, month; yr, year; AIS, acute ischemic stroke; CI, confidence interval; PD, Parkinson's disease; TIA, transient ischemic attack.

### 3.3 Frailty, Vascular Comorbidities, and Military‐Relevant Risk Factors

A secondary analysis was performed following propensity matching for frailty (Fig. 1B), as the VA frailty index was significantly different between the four main groups (Table 1), and frailty is known to associate with mortality. Like before, the cumulative incidence of death was highest in the PD + stroke group. When restricting to the dementia‐based codes of the frailty index, the significant synergy observed in PD + stroke persists (Supplementary Table S3, Fig. S4). Next, matching for common vascular comorbidities was performed as groups showed differences in their baseline prevalence (Table 1), with the PD + stroke group demonstrating numerically higher rates of all risk factors except atrial fibrillation. These data recapitulated the original findings, suggesting synergism in mortality in the PD + stroke group (Fig. 1C). To further understand whether statin exposure before or 90 days after index date may have confounded the relationship between stroke and mortality, matching for statins was performed and the primary result was recapitulated (Supplementary Tables S4 and S5, Fig. S5). Cumulative incidence of death at 10 years was 63% in the control group, 71% in the stroke‐only group, 68% in the PD‐only group, and 86% in the PD + stroke group. A secondary analysis with propensity‐matching for both PTSD and TBI was performed, which also recapitulated our primary result (Fig. 1D). Cumulative incidence of death at 10 years was 57% in the control group, 69% in the stroke‐only group, 65% in the PD‐only group, and 85% in the PD + stroke group. Finally, repeating these analyses when deploying the high PPV definition of PD confirmed these findings (Supplementary Fig. S1A–D, Table S1).

### 3.4 Stroke Characteristics

**FIG. 2:**
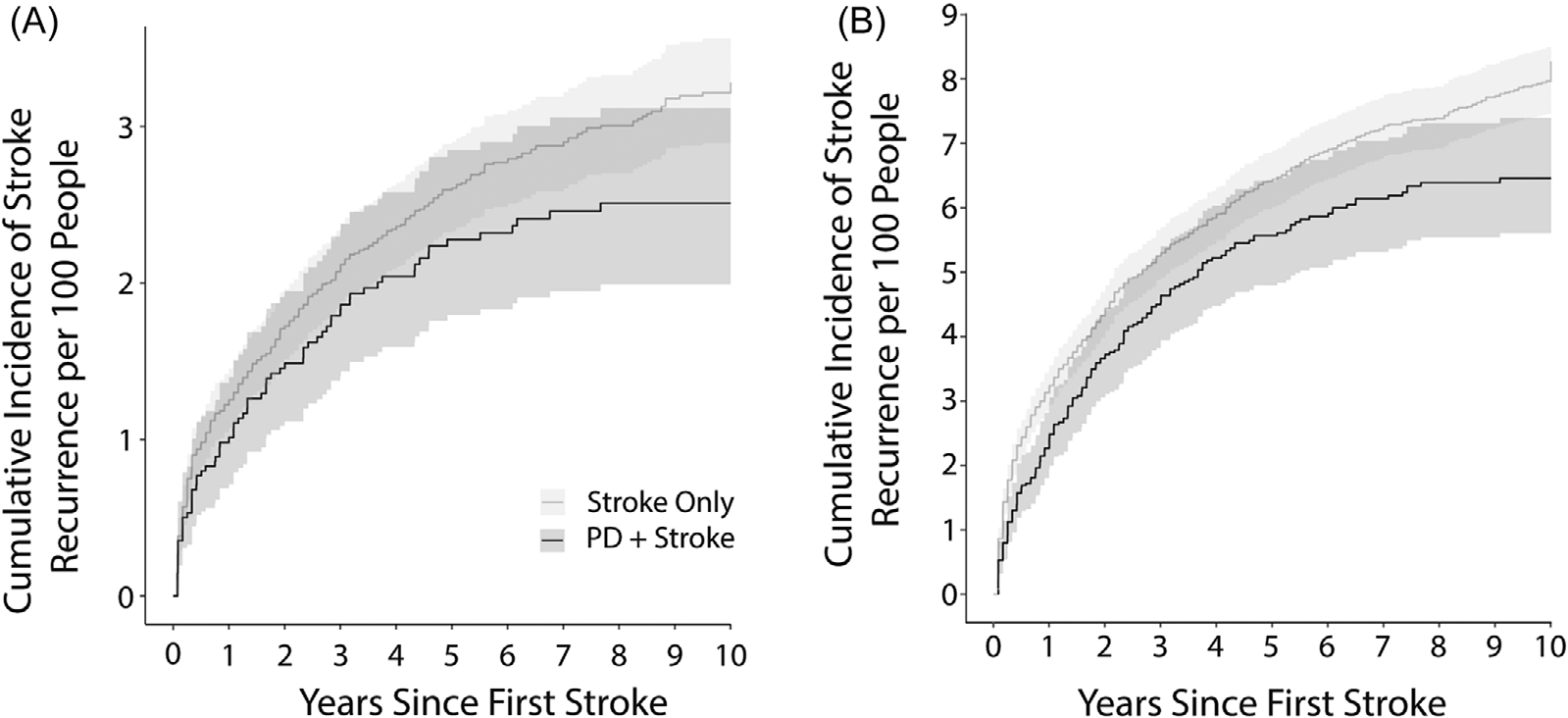
Incidence of stroke recurrence in relation to Parkinson's disease (PD). Cumulative incidence of stroke recurrence adjusted for competing risk of death in patients with stroke‐only (light gray) and PD + stroke (dark gray). (A) Stroke recurrence is defined as the presence of new inpatient stroke International Classification of Diseases (ICD) code occurring after coded inpatient discharge from initial stroke. (B) Stroke recurrence is defined as any inpatient stroke ICD code greater than 30 days after the initial stroke.

**FIG. 3:**
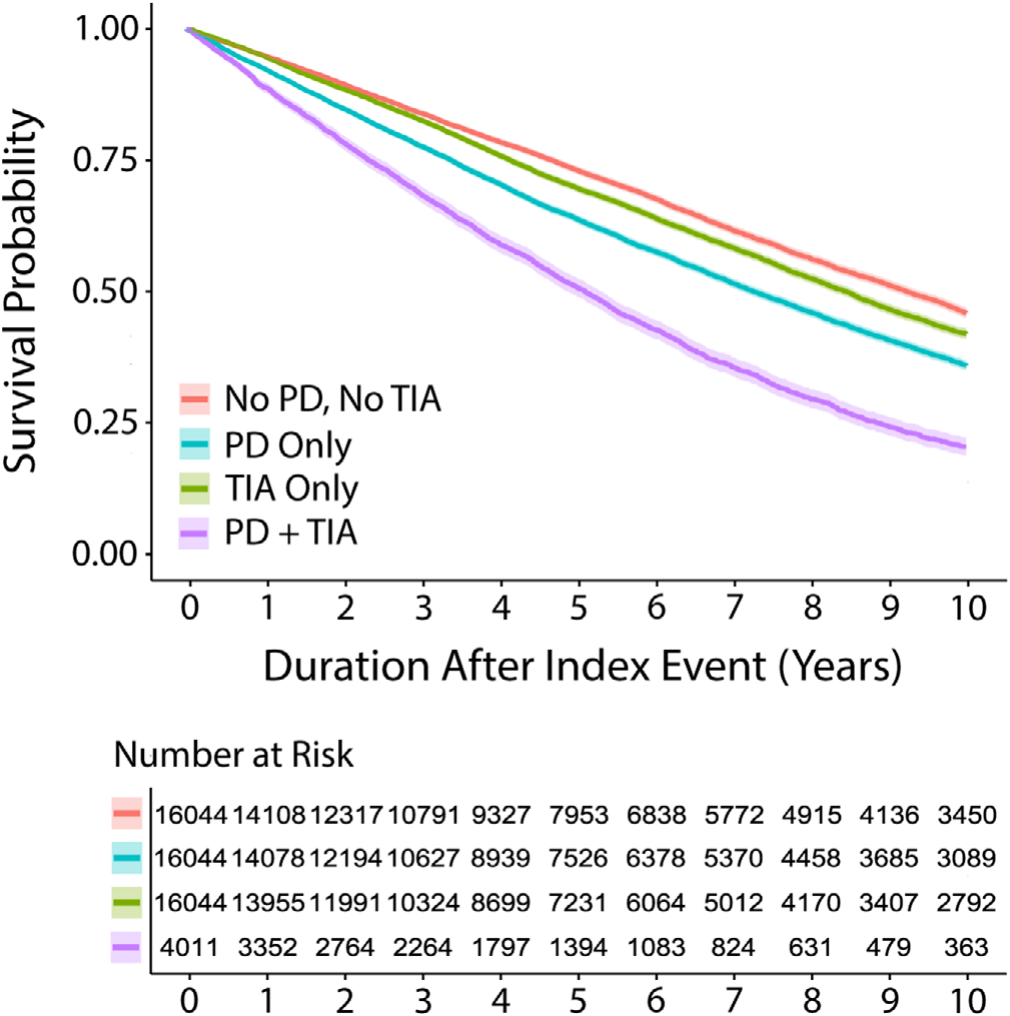
Transient ischemic attack (TIA) survival curve. Survival probability is graphed for 10 years after the index date (date of TIA or equivalent age in controls) for groups: Parkinson's disease (PD) + stroke (purple), PD‐only (cyan), stroke‐only (green), and controls (red). Survival probability is adjusted for sex, race, and smoking status. Risk tables for each group at yearly intervals are included under the graph. [Color figure can be viewed at wileyonlinelibrary.com]

Among the PD + stroke and stroke‐only cohorts, 15,865 individuals experienced an AIS. Of the random subset manually reviewed, the majority of these strokes (72%) were classified as small, but they were broadly distributed throughout the brain with no predilection for any one region (Supplementary Table S1). When comparing those veterans with and without pre‐existing PD, there were no significant differences in presumptive mechanism or regional distribution; however, the stroke‐only group had significantly more medium‐ and large‐sized strokes compared with the PD + stroke group (36.8% vs. 20.0%, *P* = 0.03). There was no difference in recurrent stroke when accounting for competing risk of death using either the more‐ or less‐restrictive definitions (Fig. 2). There were 4011 cases identified with PD + TIA. In contrast to AIS, there was a smaller decrease in 10‐year survival between the TIA‐only and control groups (57% vs. 53% mortality) (Fig. 3). The results were recapitulated when deploying the high PPV definition of PD (Supplementary Fig. S2). Survival analysis showed slightly higher mortality at two later time intervals (Table 2). Strikingly, PD + TIA showed greater overall mortality (78%) compared with PD‐ or TIA‐only, with additional mortality starting earlier (by 6 months after the index event) and persisting into later timepoints (Table 2). However, the cumulative PD‐TIA mortality was lower than that from PD + stroke.

## 4 Discussion

This retrospective cohort study of US veterans uncovers a potent and long‐lasting interaction between PD and AIS of substantially increased mortality – beyond what is expected from either condition alone. This was unexplained by differences in stroke recurrence, traditional vascular risk factor burden, comorbid neurotrauma, and stroke severity. In fact, those with PD + stroke had a statistically significantly greater proportion of smaller infarcts compared with those with stroke‐only.

Whereas some prior studies have shown an increased mortality rate in those with both PD and AIS,^4^, ^7^, ^8^ a major strength of this study is the mitigation of misclassification by deploying a previously validated algorithm to ensure high specificity of PD which minimizes mimics that may represent entirely different neuropathologies. In addition, replication of our sensitivity analyses using alternative definitions and different statistical methods further supports our main findings. An added strength is the inclusion of PD‐only and stroke‐only groups to disentangle their individual effects from interactions between them. Also unique to this study is the nuanced timing information. These data suggest a steep increase in mortality in the early and intermediate time periods before mirroring each pathology alone at later stages.

Unexpectedly, the current study does not implicate the canonical vascular risk factors as contributory to the synergy between PD and stroke. Thus, these pathways may not, in isolation, be long‐term treatment targets for addressing mortality. However, while this study examined rates of comorbidities, it did not evaluate adherence to treatment for addressing these comorbidities. Differences in adherence patterns may have contributed to unmeasured differences in structural networks. Adherence to guideline‐directed therapy might be similar, as following the index stroke there were no differences in stroke recurrence following the index stroke. Another possibility is that stroke itself induces cerebrovascular dysfunction,^22^ which compounds the pathological burden on individuals suffering from PD. This has been observed in humans as well as in rodent models of AIS.^23^ Thus, the main finding highlights the need for new approaches to treat PD and stroke since these are common conditions with negative consequences and tailored treatment has benefited other at‐risk groups (eg, sickle‐cell disease, mental health disorders).^3^, ^24^, ^25^ In this regard, interventions targeting the downstream vascular dysfunction triggered by stroke can be a productive avenue. Alternatively, more aggressive screening in those with underlying PD even 5 years post‐stroke for complications such as dysphagia may be warranted, which is not a strong emphasis in current practice.^26^

Due to the nature of the study design, the question of true incidence of stroke seen in this subpopulation of people with PD cannot be answered. However, we were able to show for the first time that stroke recurrence was no different between these two groups. Two studies from China^5^ and Taiwan^4^ suggested the incidence of stroke among those with PD was approximately two‐fold higher than those without. In contrast, a smaller Canadian study drew the opposite conclusion after appropriately adjusting for smoking status^27^ and a meta‐analysis of four pathological studies revealed a modestly significant odds ratio that approached a value of 1.^6^ The present data on recurrence argue against more severe or frequent strokes underlying this interaction between PD and stroke since it does not fit the observation of decade‐long ongoing synergy.

These data also do not support the idea that PD directly changes the mechanism or severity of stroke. While others have inferred causal links between PD and cardioembolic strokes,^28^, ^29^, ^30^ clinicopathological studies have suggested either no difference in infarct frequency and mechanism^31^ or a higher prevalence of lacunar strokes.^32^ While our results do not indicate any predilection for stroke subtype, we do show that those with PD are more likely to experience smaller strokes. In light of this lack of difference, it is difficult to recommend practice changes. Future studies are needed to understand the neurochemical microenvironment and native vascular properties in PD, which may explain why individuals with PD exhibited smaller strokes.

The data do not elucidate a mechanism, but some speculation is possible. Some authors have argued that dopamine deficiency in PD may protect these patients from dopamine‐mediated excitotoxicity.^27^, ^33^ However, more recent preclinical work by Lohmann et al. has shown that middle cerebral artery occlusion in mice overexpressing the A53T mutant of human α‐synuclein leads to a biphasic inflammatory response, one early and one late, which parallels the increasing aggregated α‐synuclein.^34^ α‐Synuclein, which may contribute to pericyte activation and vascular destabilization,^35^ is increased after stroke, and targeted reduction of pathological α‐synuclein reduces infarct size, improves stroke survival, and improves functional recovery in animal models.^36^ Therefore, medications targeting α‐synuclein may show potential in improving long‐term outcomes. Other groups have shown widespread bioenergetic insufficiencies in areas beyond the ischemic core.^22^ Still others have shown it is not dopamine deficiency per se, but rather the impairment of PD‐relevant genes such as DJ‐1 leading to increased sensitivity to excitotoxicity and ischemia.^37^ To that end, a plausible treatment target would be to augment dopaminergic signaling through carbidopa‐levodopa administration. At least in a stroke population without pre‐existing PD, a large randomized controlled trial of carbidopa‐levodopa paired with standard therapy failed to show benefit in mobility, mood, cognition, or mortality at 8 weeks or 1 year.^38^ Differential rates of levodopa administration in the PD + stroke group were not analyzed, and so it is unknown whether this could explain the difference in outcomes. In fact, it is known that many people with PD do not receive their appropriate doses of medication at the appropriate intervals.^39^, ^40^ While this may contribute to the acute mortality difference, it is unlikely to influence mortality differences 5 years after the index stroke.

An alternative hypothesis argues for differences in vasculature. It has previously been shown that in patients with PD, as compared with age‐matched controls, the cerebral blood vessels are shorter, wider, sparser, and with less arborization.^41^ The downstream consequences are to compromise oxygen delivery and render the brain more susceptible to ischemic insults. Compounding this poor substrate is the high rate of orthostatic hypotension (OH). An over 30% cross‐sectional prevalence of OH has been reported in PD,^42^ with that number increasing dramatically as the disease progresses.^43^ More troublesome is that in a population of people with rapid eye movement (REM) sleep behavior disorder – a prodromal population where synuclein pathology is likely to have initiated but motor symptoms remain latent – perhaps as many as 27% meet the criteria for OH.^44^ It has been shown that people with OH demonstrate autoregulatory failure – when brought upright on a tilt table the blood flow velocities of their middle cerebral artery dropped inappropriately.^45^ A recent prospective study has borne this out, where those with OH have a two‐fold greater risk of incident stroke compared with those without OH.^46^ Thus, while aggressive blood pressure control has been shown to confer benefit in a broad population, it is unknown if this may be deleterious in people with OH and supine hypertension.

This study is not without limitations. First, the results from this study may not generalize, as non‐White non‐male individuals are underrepresented and only US veterans were included. Although sample sizes were sufficient to detect effects of other demographic subpopulations, there is still a risk of bias, particularly of underrepresented intersectional individuals such as non‐White females. This is critical to study since female sex and some non‐White races confer increased stroke mortality risk. Second, while comorbidities thought to be military‐specific (eg, PTSD) may be overrepresented in this sample, a recent systematic review suggested lifetime prevalences of PTSD among civilians and veterans are similar.^47^ Moreover, PTSD is a recently established risk factor for stroke,^48^ even among civilians,^49^ and an important factor to consider in epidemiological studies as it is found in higher rates post‐stroke and contributes to worse outcomes.^25^, ^50^ Third, while vascular risk factors were accounted for and acute symptomatic strokes were evaluated in a small subset of this sample, future studies examining the possible contribution of silent vascular events is warranted.

Furthermore, in electronic health record‐based cohort studies such as this one, several relevant covariates are unable to be captured including genetic background, dietary habits, exercise, and sleeping patterns.^51^, ^52^ Entities such as dietary and exercise patterns are prohibitively laborious to curate for a study of this size, but smaller follow‐up studies should examine this because these factors prevent and treat both PD and stroke as well as radiographic neurovascular disease.^53^, ^54^ Of particular interest is homocysteine, an unexamined factor due to substantial data incompleteness. Several studies have reported that people with PD have measurably higher levels of homocysteine compared with healthy controls,^55^ and this difference becomes exaggerated following chronic treatment with levodopa,^56^ where it is associated with worsening non‐motor symptoms such as cognitive changes.^57^ Likewise, homocysteine is considered an independent risk factor for stroke^58^ and vascular dementia.^59^ Homocysteine may contribute to the pathogenesis of both diseases by promoting inflammation, enhancing oxidative stress,^60^ and accelerating atherogenesis.^61^ Furthermore, in preclinical models, homocysteine has also been shown to disrupt brain function by post‐translationally modifying proteins, directly activating neurotransmitter receptors, and by inducing vascular impairments such as blood–brain barrier dysfunction, microhemorrhages, and decreased cerebral blood flow.^59^ Homocysteine levels are also linked to a higher rate of distributive network problems in patients with PD and striatal silent lacunar infarctions as suggested by a much greater degeneration within the substantia nigra, which predicted greater motor disability and greater need for dopaminergic therapy 1 year later.^62^ Therefore, elevated homocysteine is closely associated with cognitive impairment^63^ and mortality,^64^ although whether, and how, it contributes specifically to the synergistic negative impact of PD and stroke is not known. Further lingering possibilities that could underlie this synergy include either an untested comorbidity – which may be considered unlikely since 31 unique diagnoses are captured by the frailty index – or differential adherence to secondary stroke prevention measures (eg, antithrombotic agents) – also considered unlikely due to the observed differential rates in comorbidities. Nevertheless, direct measurement could resolve this issue.

## 5 Conclusions

US veterans with both PD and stroke demonstrate significantly higher mortality rates than patients with either condition alone, despite fewer comorbidities, lower recurrence rates, and smaller infarct sizes. These data support prior work showing structural and network dysfunction and may lead to targeted efforts to address the deleterious impact of cerebrovascular disease in those with PD.

## Author Roles

(1) Research Project: A. Design, B. Execution, C. Data Analysis, D. Supervision; (2) Statistical Analysis: A. Design, B. Data Analysis, C. Review and Critique; (3) Manuscript Preparation: A. Writing of the First Draft, B. Editing of the Final Version.

L.E.N.: 1C, 2A, 3A, 3B.

C.L.: 1A, 1C, 3B.

A.M.: 1C, 3B.

G.D.S.: 1A, 1B, 1D, 2A, 2B, 3B.

## Supporting information


**Data S1.** Supporting Information.

## Data Availability

The data that support the findings of this study are available on request from the corresponding author. The data are not publicly available due to privacy or ethical restrictions.
