# Supplementary material for: Small Strokes, Big Impact: Excessive Mortality After Acute Ischemic Stroke in Parkinson's Disease
Source: Mov Disord. 2026 Feb 9;41(5):1141–51. doi: 10.1002/mds.70218 (PMC13193710; doi:10.1002/mds.70218)
Supplement: Supplementary file 1 — Data S1. Supporting Information. [file MDS-41-1141-s001.docx]

## Supplementary Figure 1. Impact of PD on survival after acute ischemic stroke when using a VA-specific definition of PD with high positive predictive value. Survival probability is graphed for ten years after the index date (date of stroke or equivalent age in controls) for groups: PD+stroke (purple), PD-only (cyan), stroke-only (green) and controls (red). Panel A shows the main analysis of survival probability adjusting for sex, race, and smoking status. The subsequent panels show additional propensity matching for comorbidities: B) frailty; C) vascular comorbidities (hypertension, hyperlipidemia, coronary artery disease, heart failure, atrial fibrillation, diabetes mellitus); and D) post-traumatic stress disorder and traumatic brain injury. Bottom: risk tables for each group at yearly intervals. A correction to time zero is needed because – by definition – patients cannot die until after their second confirmatory VA neurology note. Thus, time zero for PD in the high PPV definition is when the second neurology note occurs and are appropriately re-matched against controls.

**
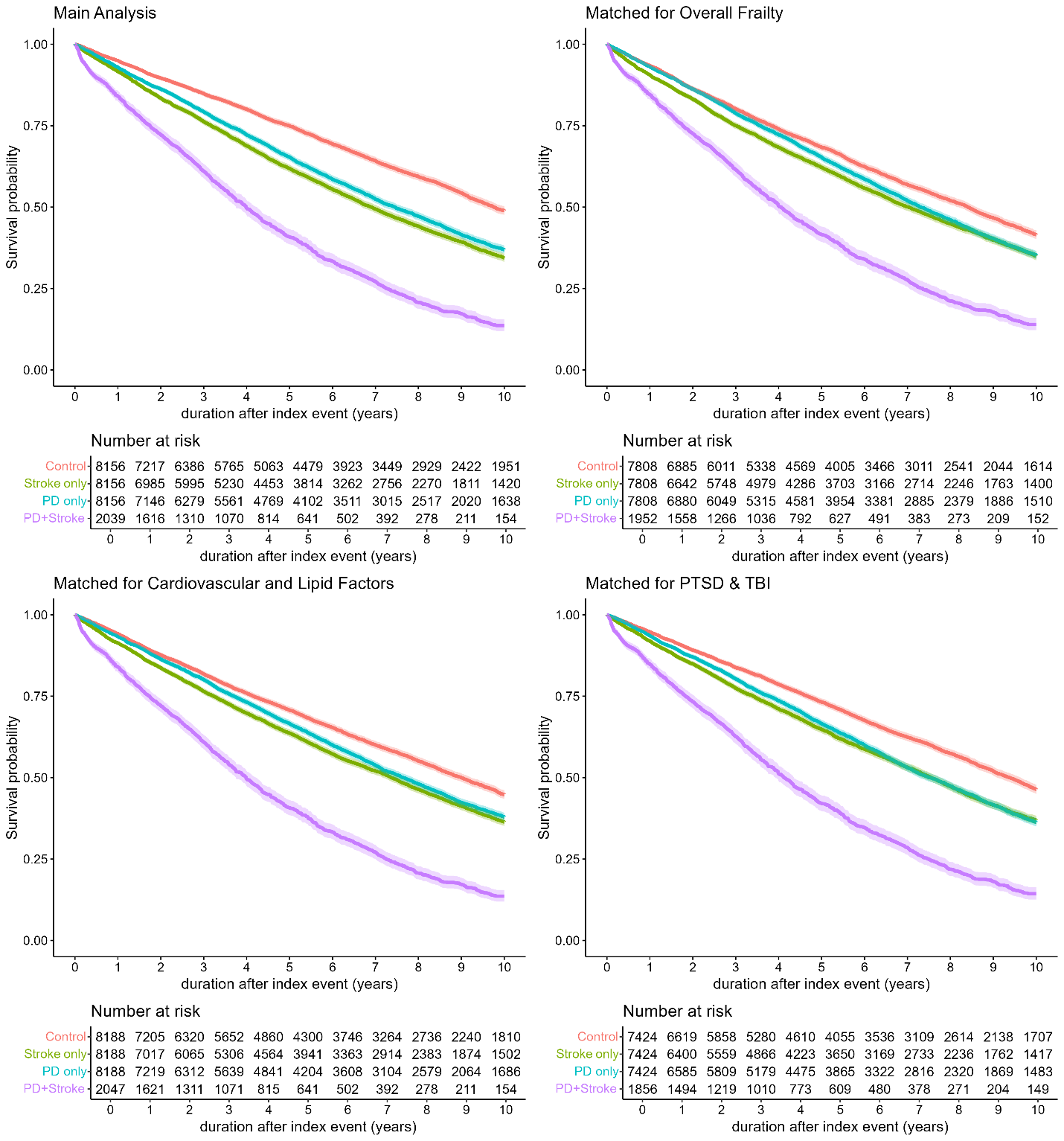
**

## Supplementary Table 1. Death due to Parkinson’s Disease (using higher PPV VA-specific PD definition), stroke, transient ischemic attack, and interactions.

|  | | Time interval | | | | | |  |  |
| --- | --- | --- | --- | --- | --- | --- | --- | --- | --- |
|  | | 0-2 months | 2-4 months | 4-6  months | 6-12 months | 1-3  years | 3-5  years | 5-10  years |  |
|  | Acute Ischemic Stroke (AIS)  # added deaths per 100 person years [95% CI] | | | | | | | | |
| PD-only | | 1.9 | -- | 2.7 | 2.7 | 2.5 | 4.0 | 4.1 |  |
|  |  | *[0.2, 3.6]* | *[-0.3, 3.3]* | *[0.6, 4.8]* | *[1.5, 3.8]* | *[1.8, 3.1]* | *[3.2, 4.8]* | *[3.3, 4.8]* |  |
| Stroke (AIS)-only | | 5.1 | 3.6 | 3.0 | 3.4 | 3.5 | 4.4 | 3.6 |  |
|  |  | *[3.1, 7.1]* | *[1.6, 5.6]* | *[0.8, 5.1]* | *[2.2, 4.6]* | *[2.9, 4.2]* | *[3.6, 5.2]* | *[2.9, 4.4]* |  |
| Additional deaths from  interaction of PD and Stroke | | 20.0 | 8.1 | -- | -- | 4.5 | 6.9 | 8.4 |  |
|  |  | *[13.5, 26.5]* | *[2.8, 13.5]* | *[-1.8, 8.4]* | *[-0.7, 4.9]* | *[2.8, 6.3]* | *[4.5, 9.3]* | *[6.0, 10.8]* |  |
| Transient Ischemic Attack (TIA) | | | | | | | |  |  |
| TIA-only | | -- | -- | -- | 2.1 | -- | -- | 2.3 |  |
|  |  | *[-5.2, 0.3]* | *[-5.5, 0.05]* | *[-5.8, 0.4]* | *[0.4, 3.8]* | *[-0.2, 1.7]* | *[-0.2, 2.4]* | *[1.1, 3.6]* |  |
| Additional deaths from  interaction of PD and TIA | | -- | -- | -- | -- | 3.0 | 3.6 | 7.5 |  |
|  |  | *[-2.4, 10.2* | *[-1.7, 11.5]* | *[-1.6, 11.7]* | *[-1.5, 7.2]* | *[0.5, 5.5]* | *[0.4, 6.8]* | *[3.8, 11.4]* |  |
|  | Abbreviations: CI = confidence interval, “*--*" = not-significant (p > 0.05); all other values significant at p < 0.05 | | | | | | | | |

## Supplementary Figure 2. TIA Survival Curve when using a VA-specific definition of PD. Survival probability is graphed for ten years after the index date (date of TIA or equivalent age in controls) for groups: PD+stroke (purple), PD-only (cyan), stroke-only (green) and controls (red). Survival probability is adjusted for sex, race, and smoking status. TIA: transient ischemic attack.

**
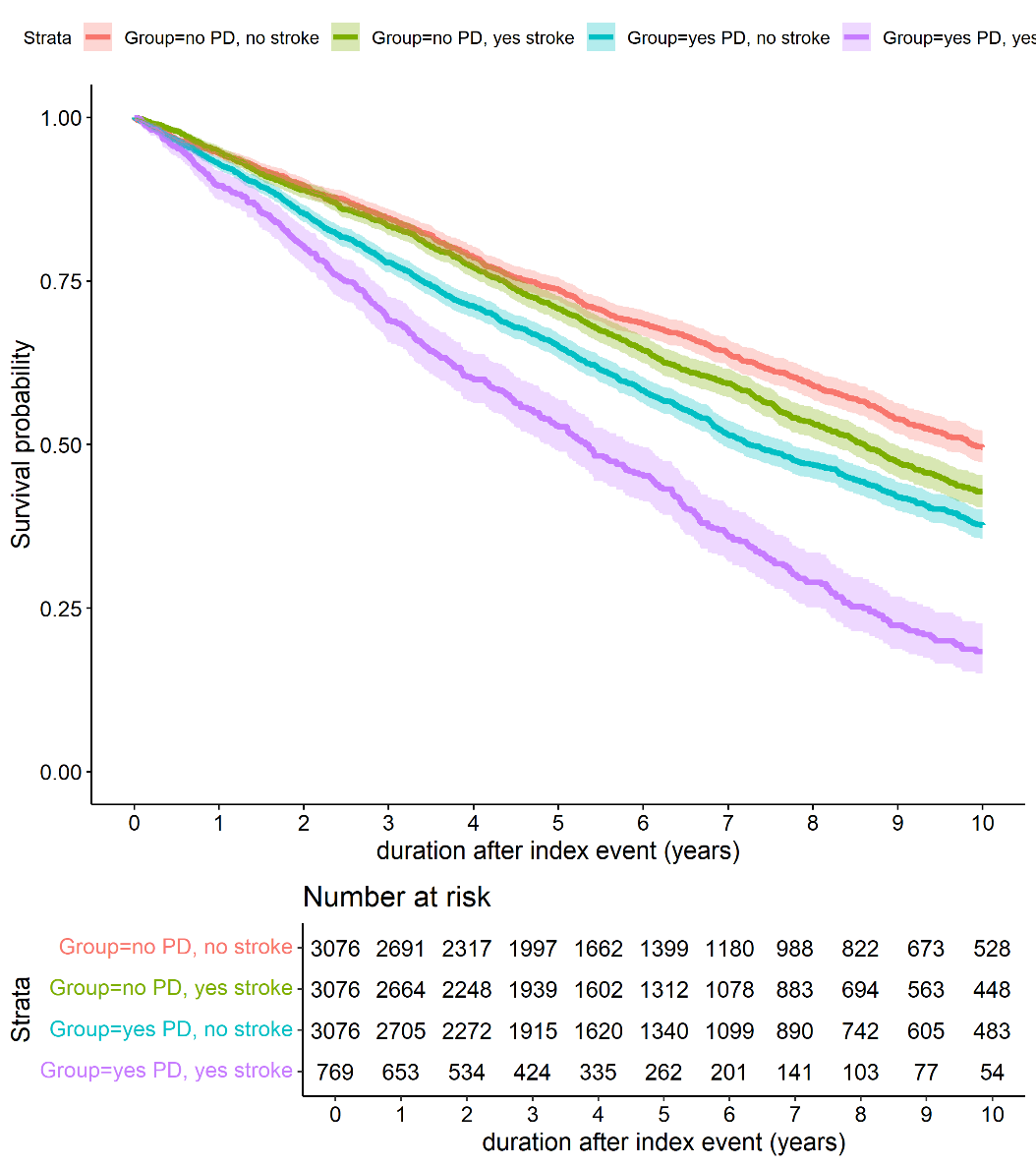
**

## Supplementary Figure 3. Impact of PD on survival after acute ischemic stroke when analysis is limited to females. Survival probability is graphed for ten years after the index date (date of stroke or equivalent age in controls) for groups: PD+Stroke (purple), PD-only (cyan), stroke-only (green) and controls (red). Panel A shows the main analysis of survival probability adjusting for sex, race, and smoking status. Panel B shows analysis when using the VA-specific, high PPV definition of PD. Bottom: risk tables for each group at yearly intervals.


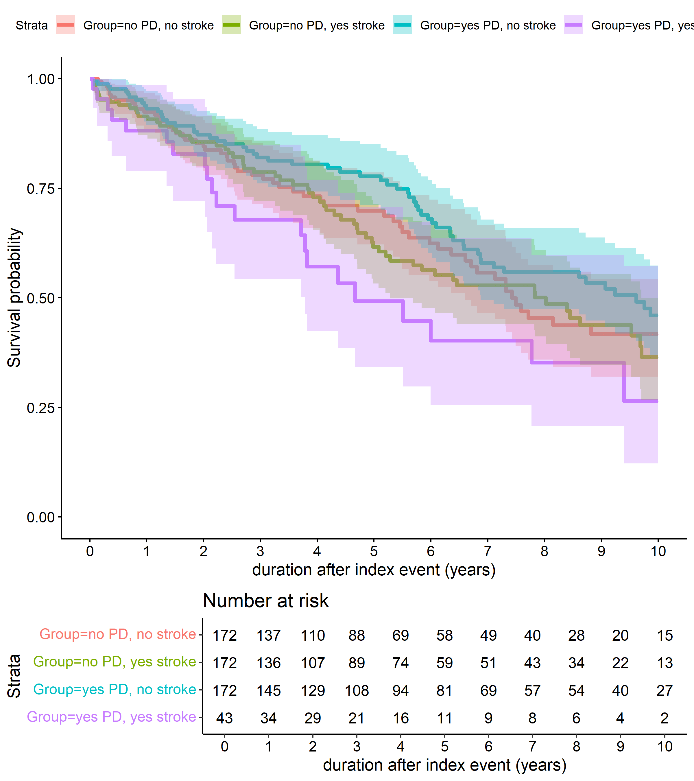

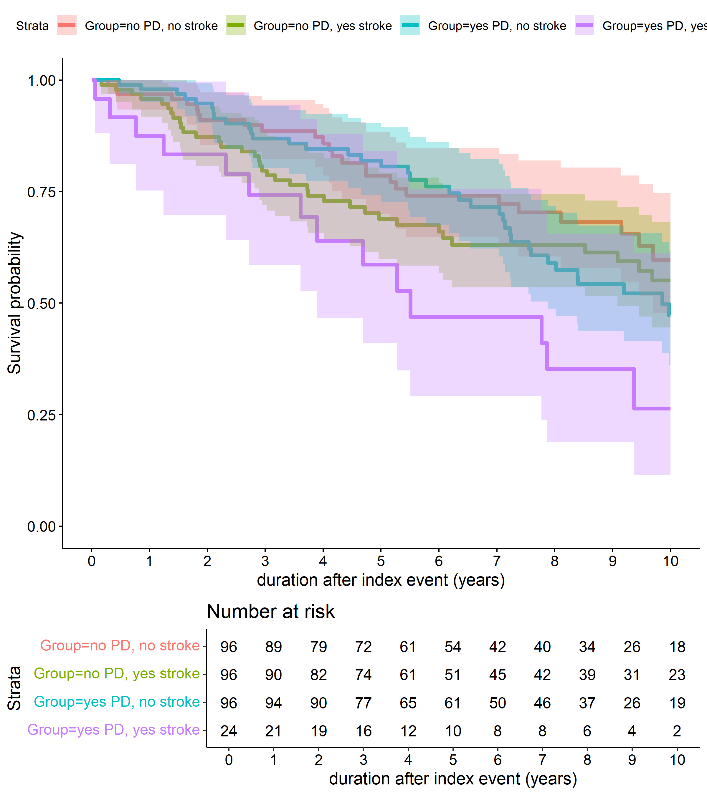


**Supplementary Table 2. Stroke Characteristics**

|  | Stroke-only | | PD+Stroke | | P Value |
| --- | --- | --- | --- | --- | --- |
| **Mechanism** |  | | | | |
| Total | 97 | 100.00% | 128 | 100.00% | 0.26 |
| Embolic | 44 | 45.36% | 49 | 38.28% |  |
| Large Artery Atherosclerosis | 15 | 15.46% | 17 | 13.28% |  |
| Lacunar | 32 | 32.99% | 59 | 46.09% |  |
| Other | 4 | 4.12% | 2 | 1.56% |  |
| Multiple etiologies | 2 | 2.06% | 1 | 0.78% |  |
| **Location** |  | | | | |
| Total | 98 | 100.00% | 99 | 100.00% | 0.24 |
| Cortex | 45 | 45.92% | 37 | 37.37% |  |
| Subcortex | 8 | 8.16% | 8 | 8.08% |  |
| Basal ganglia | 26 | 26.53% | 32 | 32.32% |  |
| Brainstem | 6 | 6.12% | 5 | 5.05% |  |
| Cerebellum | 13 | 13.27% | 17 | 17.17% |  |
| **Size** |  | | | | |
| Total | 87 | 100.00% | 95 | 100.00% | **0.03** |
| Large | 7 | 8.05% | 6 | 6.32% |  |
| Medium | 25 | 28.74% | 13 | 13.68% |  |
| Small | 55 | 63.22% | 76 | 80.00% |  |
| Data are n (% of total). The ‘Other’ mechanisms include periprocedural stroke (4), aortic arch atheroma (1), and hypercoagulable state (1). | | | | | |

**Supplementary Table 3. Cohort characteristics stratified by cognitive status.**

Dementia was defined using the VA frailty ICD and CPT-based definition of dementia including ICD9 codes (290, 290.[0-4], 291.[1-2], 293.1, 294.[8-9], 331, 331.[11,82,83], 438, 780.[09,93], 799.5), ICD10 codes (F01.5[1,2], F02.8[0-1],F03.9[0-1], F04x, F05x, F06.[0,8], F10.[26-27,96-97], G30.[0,1,8,9], G31.0[1,9], G31.[1,2,83,84], G94x, I69.01x, I69.11x, I69.21x, I69.31x, I69.81x, I69.91x, R40.[0-1], R41.[1-3], R41.[81,84x,89], R54x), and CPT codes (1490F, 1491F, G0505, 1493F).

| Group | Control  no PD, no stroke | Stroke only  (no PD) | PD Only  (no stroke) | PD + Stroke |
| --- | --- | --- | --- | --- |
| N | 12692 | 12692 | 12692 | 3173 |
| Dementia: Before Matching | 1,796 (14.2%) | 3,876 (30.5%) | 5,700 (44.9%) | 1,833 (57.8%) |
| Dementia: After Matching | 4,515 (39.8%) | 4,452 (39.3%) | 4,326 (38.2%) | 1,132 (40.0%) |

## Supplementary Figure 4. Impact of PD on survival after acute ischemic stroke when matching for dementia. Survival probability is graphed for ten years after the index date (date of stroke or equivalent age in controls) for groups: PD+stroke (purple), PD-only (cyan), stroke-only (green) and controls (red). The left panel reproduces main figure 1A (the main analysis of survival probability adjusting for sex, race, and smoking status). The right panel shows additional propensity matching for dementia. *Bottom: risk tables for each group at yearly intervals*.


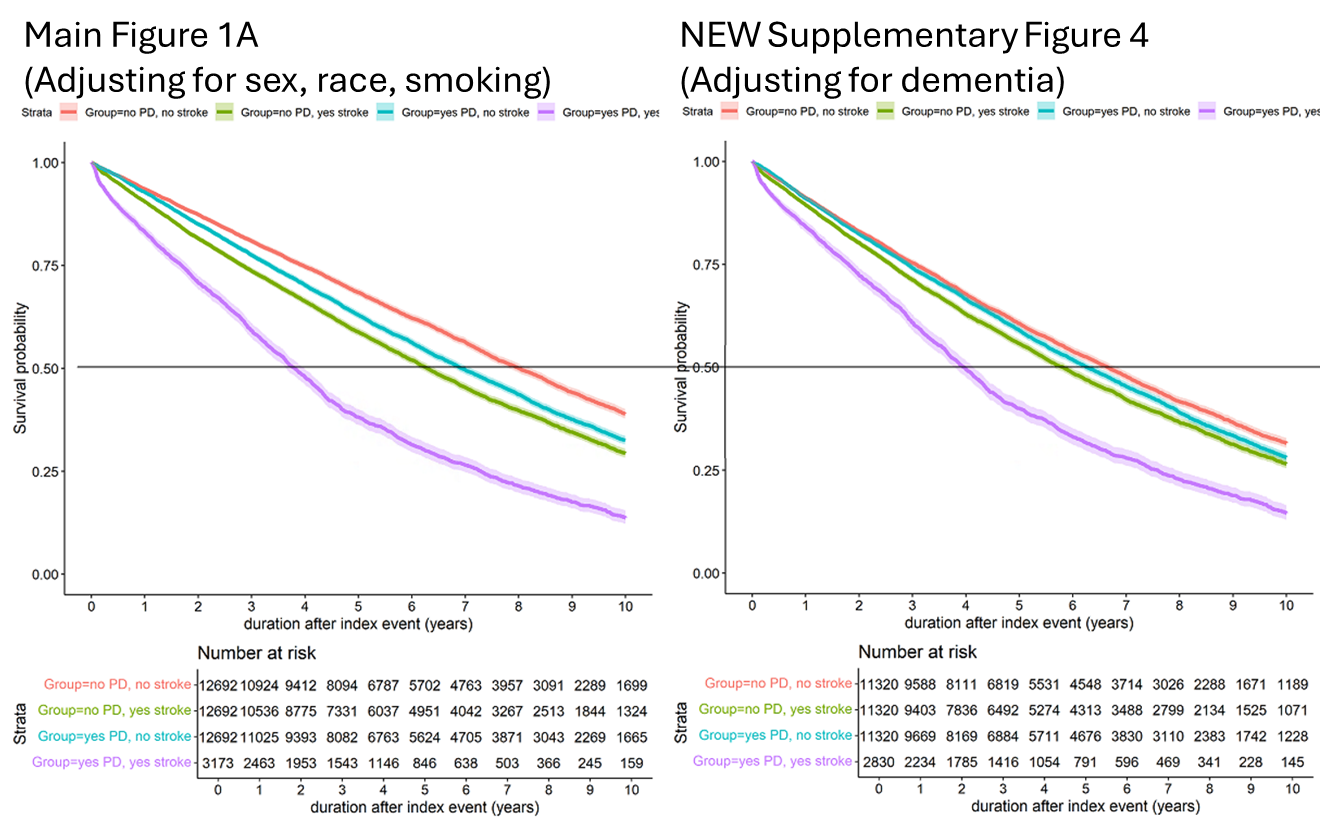


**Supplementary Table 4. Cohort characteristics stratified by statin use.**

Statin use – defined as a minimum of 2 recurrent prescriptions for atorvastatin, lovastatin, pravastatin, rosuvastatin, and/or simvastatin BEFORE index date OR 1+ prescription from 90 days AFTER index date – is recorded in the table for all groups. When matching is performed, the groups are ALSO matched for age, sex, and smoking status.

| Group | Control  no PD, no stroke | Stroke only  (no PD) | PD Only  (no stroke) | PD + Stroke |
| --- | --- | --- | --- | --- |
| N | 12692 | 12692 | 12692 | 3173 |
| Statins: Before Stroke | 4413 (34.8%) | 6782 (53.4%) | 7777 (61.3%) | 2231 (70.3%) |
| N | 11904 | 11904 | 11904 | 2976 |
| Statins: Before Stroke (matched) | 8449 (71.0%) | 8335 (70.0%) | 8347 (70.1%) | 2113 (71.0%) |
| N | 12216 | 11986 | 12262 | 2919 |
| Statins: After Stroke | 1293 (10.6%) | 4514 (37.7%) | 2204 (18.0%) | 1020 (34.9%) |
| N | 6132 | 6132 | 6132 | 1533 |
| Statins: After Stroke (matched) | 1047 (17.1%) | 1018 (16.6%) | 1068 (17.4%) | 267 (17.4%) |

## Supplementary Figure 5. Impact of PD on survival after acute ischemic stroke when matching for statins. Survival probability is graphed for ten years after the index date (date of stroke or equivalent age in controls) for groups: PD+stroke (purple), PD-only (cyan), stroke-only (green) and controls (red). Panel A depicts cumulative survival curves when groups are matched on statin use BEFORE the index date (in addition to adjusting for sex, race, and smoking status). Panel B depicts cumulative survival curves when groups are matched on statin use 90 days AFTER index date (in addition to adjusting for sex, race, and smoking status). *Bottom: risk tables for each group at yearly intervals*. Note: In panel B, the curve is necessarily flat in the beginning because no one can die until 90 days after index date.


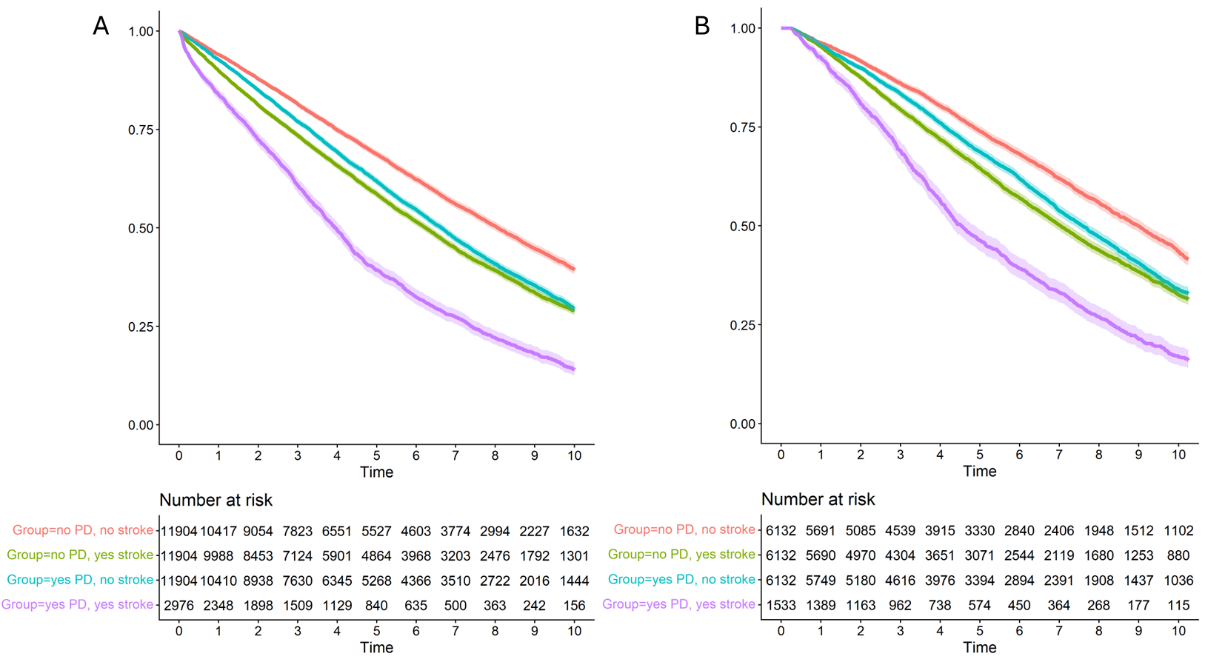


## Supplementary Table 5. Death due to Parkinson’s disease, stroke, and interactions when matched for statin use.

|  | Time interval | | | | | | |
| --- | --- | --- | --- | --- | --- | --- | --- |
|  | 0-2 months | 2-4 months | 4-6  months | 6-12 months | 1-3  years | 3-5  years | 5-10  years |
| Additional deaths from interaction of PD and Stroke MATCHED starting BEFORE index date | 16.7  *[11.3, 22.1]* | 7.1  *[2.5, 11.7]* | -- | -- | 3.6  *[2.2, 5.1]* | 8.5  *[6.3, 10.6]* | 3.2  *[1.2, 5.3]* |
|  | 0-3 months | 3-4 months | 4-6 months | 6-12 months | 1-3 years | 3-5 ears | 5-10 years |
| Additional deaths from interaction of PD and Stroke MATCHED starting 90 days AFTER index date | N/A | 7.4  *[0.4, 14.4]* | 5.0  *[0.4, 9.7]* | -- | 3.7  *[1.9, 5.5]* | 7.6  *[5.1, 10.2]* | -- |
| Abbreviations: CI = confidence interval, “--" = not-significant (p > 0.05); all other values significant at p < 0.05 | | | | | | | |
